# *Ex situ* thermal acclimation alters temperature selection with limited post-release effects in an endangered frog (*Rana muscosa*)

**DOI:** 10.1098/rsos.260332

**Published:** 2026-10-07

**Authors:** Kenzi M. Stemp, Talisin T. Hammond, Michel E. B. Ohmer, Gregory F. Grether, Debra M. Shier

**Affiliations:** ^1^ San Diego Zoo Wildlife Alliance, Escondido, CA 92027, USA; ^2^ Department of Ecology and Evolutionary Biology, University of California Los Angeles, Los Angeles, CA 90094, USA; ^3^ Department of Biology, University of Mississippi, Oxford, MS 38677, USA

**Keywords:** amphibian, chytrid fungus, conservation, disease, *ex situ* breeding, *Rana muscosa*, reintroduction, thermal acclimation, translocation

## Abstract

Amphibian declines driven by the fungal pathogen *Batrachochytrium dendrobatidis* (Bd) pose major challenges for conservation, particularly for cold-adapted species occupying habitats where temperatures may be less limiting to Bd growth. Host thermal behaviour can influence infection dynamics, and thermal environments during developmental stages may shape disease susceptibility and performance. We tested whether exposing endangered mountain yellow-legged frogs (*Rana muscosa*) to different developmental *ex situ* thermal treatments prior to a reintroduction event altered selected temperature and post-release habitat use, Bd outcomes and apparent survival. Temperature-selection trials revealed that selected temperature was moderately repeatable among individuals. Contrary to predictions, frogs reared in a high temperature treatment subsequently selected cooler temperatures than frogs reared under standard conditions. After release at a Bd-positive site, high temperature treatment frogs exhibited marginally higher short-term apparent survival. However, survival declined rapidly regardless of treatment and we found no treatment-based differences in microhabitat use or Bd patterns, possibly due to low detection rates in the field. These results demonstrate that *R. muscosa* shifts selected temperature in response to thermal acclimation during rearing, but that such shifts may not translate to improved establishment in Bd-positive environments. Optimizing reintroduction outcomes for Bd-susceptible amphibians will likely require combining husbandry-based thermal conditioning with complementary Bd mitigation strategies.

## 1. Introduction

Amphibians are experiencing unprecedented population declines [1,2] with the fungal pathogen *Batrachochytrium dendrobatidis* (Bd) implicated as a major driver of extinction risk [3–8]. Bd causes amphibian chytridiomycosis, a disease which invades keratinized structures and can rapidly impair electrolyte transport and osmoregulatory function, leading to mortality [9–12]. Host-Bd dynamics are complex and depend strongly on interactions between host physiology, pathogen traits and abiotic factors, particularly temperature [13–19].

Because Bd grows best at moderate temperatures (18–20°C) and is inhibited at higher temperatures (more than 28°C), thermal conditions shape both infection intensity and host survival [9,20,21]. Consequently, infection prevalence is typically lower at warmer environmental temperatures [15,22–25]. However, some studies suggest that a thermal mismatch between the host and its environment during infection may be a stronger predictor of mortality than temperature alone, and that hosts experience the greatest disease impacts when environmental temperatures favour pathogen performance over host physiological optima. For example, cold-adapted species may experience heightened susceptibility to Bd in warm environments, and vice versa [26,27]. Also, host immunity and behaviour can vary widely among species and individuals, which can affect disease dynamics, and Bd may be locally adapted to thermal conditions [28–31]. Therefore, it is important to consider host-pathogen dynamics and the optimal temperature ranges for both hosts and Bd strains at individual-, species- and location-specific levels.

Many physiological processes function more efficiently when an organism achieves the thermal optimum for that process [32]. As ectotherms, amphibians fine-tune their body temperature continuously by behaviourally adopting specific body postures or selecting microhabitats (e.g. basking) that allow them to regulate their thermal environment [33–35]. Within and across species, thermal preferences may affect disease outcomes [36–40], with evidence suggesting that the change in temperature from an individual's baseline is more influential in mitigating disease than reaching a specific threshold [39]. Laboratory studies suggest that holding amphibians at increased temperatures can reduce or clear Bd infections [41–43], and some amphibians implement a ‘behavioural fever’ in response to Bd by seeking out warmer microhabitats that inhibit pathogen growth, thereby reducing infection intensity [19,25,37–39]. Behavioural cooling may also be implemented as a mechanism to reduce disease impacts in one cold-adapted species [44]. Yet despite extensive work on thermal physiology and disease in amphibians, we still know relatively little about whether selected temperatures can be altered through early-life experience (but see [45]) and whether such shifts persist in natural settings.

Given the ongoing declines of amphibians globally, these taxa are increasingly a part of *ex situ* breeding and reintroduction programmes, where environmental conditions can be controlled and manipulated before individuals are released into the wild [46,47]. These programmes offer a unique opportunity to investigate thermal biology and to better understand which factors might shape selected temperature. The theory of natal habitat preference induction suggests that animals should prefer habitats similar to those in which they developed [48]. And exposure to naturalistic conditions in *ex situ* breeding facilities has proven a useful tool for increasing post-release survival [49,50]. Thermal conditions in *ex situ* facilities are often designed to mimic those that a species might experience in the wild, but only a few studies have experimentally tested how developmental temperature influences selected temperature, disease outcomes or immune function for reintroduced animals in the wild [45,51].

Physiological acclimation in temperature selection has been well studied, with evidence that many ectothermic species are able to reversibly shift temperature selection or preference when exposed to environmental variation during their lifetime [52–56]. Temperature-related life history traits can also be developmentally plastic and can result in lasting changes in thermal phenotype in ectotherms [45]. This acclimation may help buffer organisms from pathogens [56,57]. Thus, making environmental changes that lead to alterations in selected temperature could theoretically reduce host-pathogen thermal overlap and improve survival following release at sites where Bd persists.

Here, we use the southern mountain yellow-legged frog (*Rana muscosa*) as a model to examine the impact of rearing temperatures on temperature selection and post-release survival in endangered, disease-threatened amphibians. Once abundant in the mountains of California, USA [58], populations of this species have sharply declined since the 1970s‒1980s [59–61] and it is now federally and state-listed as endangered [62,63]. The southern California distinct population segment of *R. muscosa* is in critical status and is currently considered extirpated from over 90% of its historic range [64–66]. Bd is ubiquitous throughout the range of *R. muscosa* in southern California, with disease-related mortality a primary driver of continued losses [67–71]. Highly aquatic and cold-adapted species like *R. muscosa* persist in cold-water streams, where environmental temperatures may increase exposure to aquatic zoospores and provide optimal temperatures for Bd growth [72,73]. Tadpoles of these species typically overwinter as larvae for 1‒3 years and can carry asymptomatic Bd infections, allowing Bd to persist across seasons [74,75]. Identifying husbandry strategies that could improve post-release survival is of high conservation value.

We use *ex situ* manipulations of the thermal environment during juvenile development to examine whether thermal acclimation alters selected temperature and influences post-release outcomes (i.e. survival, microhabitat selection and disease status) in *R. muscosa* [41,45,76]. During juvenile development (late-stage tadpoles to 6-month-old juveniles) and prior to reintroduction, we manipulated basking temperature availability in an *ex situ* facility to mimic the temperature extremes *R. muscosa* may experience in the wild, providing conditions with variable thermal opportunity. Our goals were to characterize baseline and repeatability of temperature selection for this population, to determine if temperature selection is plastic in response to thermal acclimation, to examine the relationship between selected temperature and post-release outcomes, and to quantify post-release survival for treatment groups. We predicted that (i) selected temperature would be a repeatable individual trait, (ii) frogs exposed to a high-temperature treatment would exhibit higher selected temperatures relative to those reared under standard conditions, (iii) selected temperature would predict microhabitat use and (iv) Bd status following release, and (v) thermal conditioning would improve post-release survival. By experimentally testing the effects of *ex situ* environments on post-release outcomes, we can gain a better understanding of how to optimize rearing conditions for this species and other species impacted by this pathogen.

## 2. Material and methods

### 2.1. Study species and thermal treatments

This study used 134 recently metamorphosed *R. muscosa* from a single clutch of an *ex situ* bred population originating in the San Jacinto Mountains of California, USA. The clutch was hatched in the spring of 2020 at the breeding facility managed by the San Diego Zoo Wildlife Alliance (SDZWA). All animals were housed and maintained in an animal care facility at the University of California, Los Angeles (UCLA) following established protocols for *ex situ* rearing of the species [77].

Frogs were assigned to one of two experimental treatments during juvenile development: a high temperature intensity treatment with additional time for basking opportunity at higher temperatures (hereafter ‘High,’ mean = 20.8, s.e. = 0.27, range = 17.8°C–30.9°C), or a low temperature intensity treatment with temperature conditions equivalent to standard husbandry practices for the species (hereafter ‘Low,’ mean = 18.6, s.e. = 0.10, range 17.8°C–24.5°C; supplementary material, table S1; figure 1a). Due to the endangered status of this species, husbandry restrictions limit average air temperature in *ex situ* tanks to no more than 24°C with additional availability of a warmer basking site [77]. Therefore, we manipulated thermal opportunity rather than forcing exposure to differing thermal environments. There were three replicate tanks for each temperature treatment. Temperature treatments were initiated on 10 May 2021 and continued until release on 29 July 2021, increasing in duration and intensity through spring, late spring and summer (figure 1a). Temperature ranges were selected using known operative temperatures for similar species and temperature data collected from local habitats using field-deployed data loggers and agar frog models [78,79]. Detailed husbandry practices and descriptions of thermal treatment development are provided in the supplementary material.

**Figure 1.**
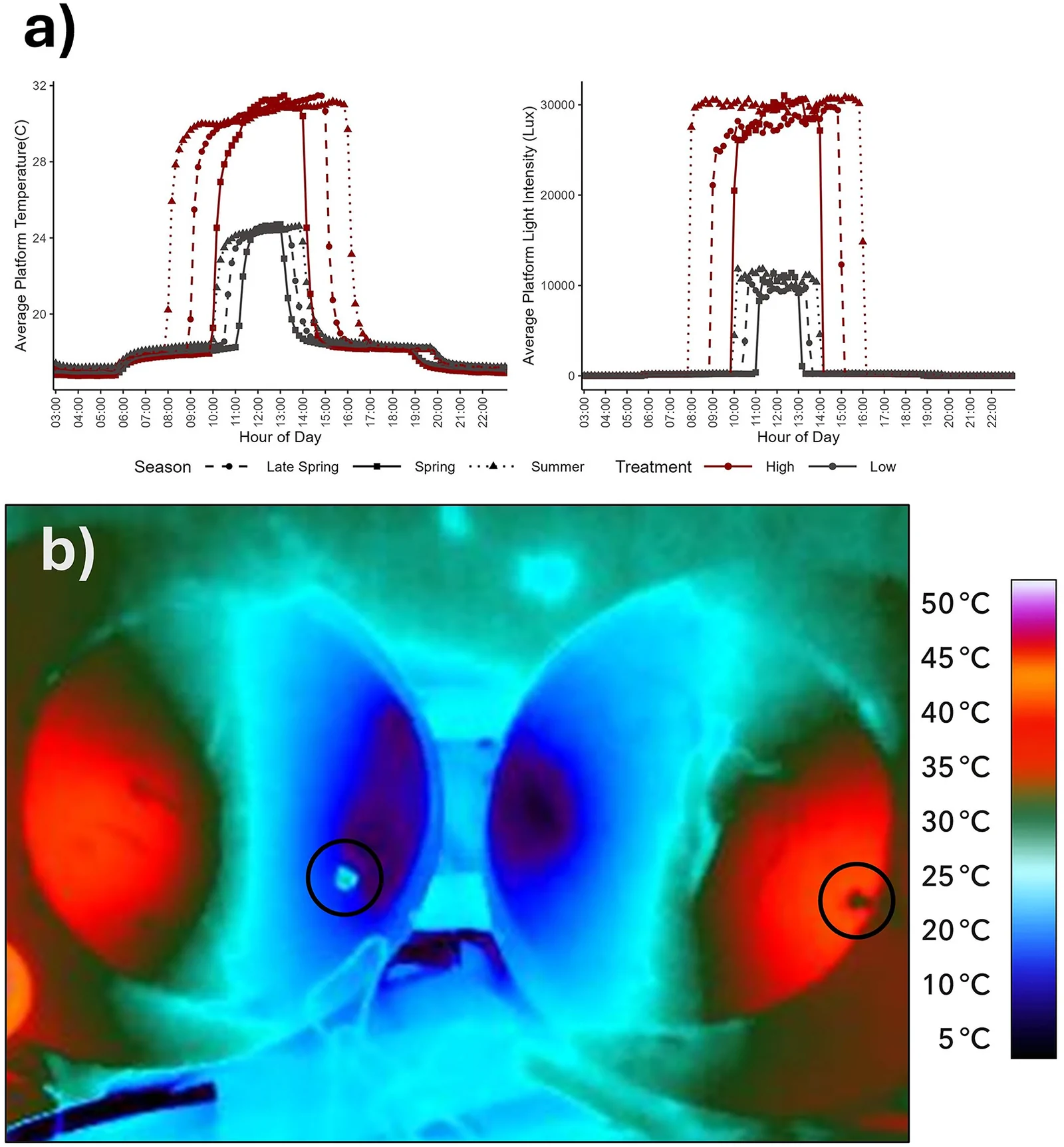
Thermal conditions (temperature and light intensity) in the high and low treatment groups during *ex situ* treatments (a) and thermal image of frogs within temperature selection trial arenas (b). Black circles indicate locations of frogs in arenas.

### 2.2. Temperature selection trials

We used established methods for amphibian temperature selection trials and a before–after-control–impact (BACI) study design to quantify selected temperature at both pre- and post-treatment timepoints [45]. In total, 130 individual frogs completed selected temperature trials (High: *n* = 66 individuals, 93 trials; Low: *n* = 64 individuals, 91 trials). Individual frogs were placed in a circular copper-coated aluminium arena (diameter = 50 cm, height = 48 cm) with a temperature gradient from approximately 10 to 30°C, representing the thermal range *R. muscosa* experiences during the active season. The gradient was created using basking lamps, heating pads and chilled water pumped through a copper pipe beneath the arena floor. Paper towels moistened with 250 ml of conditioned water lined the arena to maintain humidity. Trials lasted 2 h between 08.30 and 15.45 under ambient conditions (mean air temperature = 18.99, s.e. = 0.05°C, mean relative humidity = 65.00, s.e. = 0.59%), with animals fasted for at least 12 h before trials. Frogs were weighed and photographed before and after each trial, and following their first trial each frog received a unique Visual Implant Elastomer (VIE) tag (Northwest Marine Technologies) for individual identification.

Pre-treatment temperature selection trials were conducted from 21 April 2021 to 9 May 2021 for a subset of 54 individuals (high: *n* = 27, low: *n* = 27; supplementary material, figure S1) to establish baseline temperature selection. Post-treatment trials for 130 individuals (including those with pre-treatment trials) were conducted from 21 May to 21 July 2021 and were timed to maximize days between pre- and post-trials for frogs that were trialled twice. For frogs with two trials, the interval between trials ranged from 51 to 87 days (mean = 68.8, s.e. = 1.2 days).

Each trial was recorded remotely using a timelapse video camera (GoPro Hero 4) and a thermal camera (FLIR E8-XT Thermal Camera, Teledyne FLIR) that captured thermal images every 30 s (FLIR ResearchIR software Version 4.30.2) to characterize temperature selection within the arena. Selected temperature was defined as the mean temperature of all frames during the last 30 min of the trial, allowing acclimation to the arena for 1.5 h [45]. Selected temperature range was calculated as the interquartile range (IQR) of the mean temperature of all frames during the last 30 min of the trial [45,80–83]. Movement was quantified from x and y coordinates (in pixel units) extracted from thermal images, providing the cumulative distance moved in each trial. Trials were manually scored using FLIR Research Studio software (Teledyne FLIR, Version 2.1). Five trials in which frogs spent more than 60% of time out of contact with the gradient were excluded. Scoring methods and inter-observer reliability are described in the supplementary material, figure S2.

### 2.3. Growth and pre-release monitoring

Body mass and snout-vent-length (SVL) were recorded monthly for all metamorphosed frogs to calculate the growth rate (mm per month). Frogs larger than 35 mm SVL received a passive integrated transponder (PIT; 8 × 1.4 mm ‘Skinny’ PIT Tag, Oregon RFID) following the methods outlined in [84]. Before release, Bd swabs were collected from a subset of individuals from each holding tank to confirm that all frogs were Bd negative before release.

### 2.4. Environmental data

To characterize relationships between selected temperature, treatment and environmental conditions at the release site, we measured canopy cover, air temperature and light intensity across the transect. Movement in recently released *R. muscosa* is typically upstream [84–86]; therefore, environmental sampling spanned from approximately 150 m downstream of the lowest release pools (which were spread across approx. 100 m) to 1250 m upstream, covering the 1.5 km survey extent. Canopy cover was quantified every 10 m using hemispherical photos taken with a 180° ‘fisheye’ lens and photos were processed with the ImageJ plugin Hemispherical 2.0 [87]. HOBO temperature and light data loggers (model: UA-002-64) were deployed every 50 m along the transect from 31 July to 24 November 2021. For each frog capture, we assigned environmental values from the nearest logger or canopy photo using the shortest Euclidean distance to the capture location.

### 2.5. Release and post-release monitoring

We released 134 1-year post-metamorphosis frogs (high: *n* = 66; low: *n* = 68) at five pools in an upper tributary of the San Jacinto River on 29 July 2021. This site historically supported *R. muscosa*, though wild frogs had not been detected since 2013 [66]. Previous reintroduction efforts occurred at the site in both 2020 and 2021 [88]. Of the released frogs, 124 were PIT-tagged; the remaining 10 were too small and were individually identified through dorsal patterning.

Following release, surveys of the stream reach were conducted at least biweekly until December 2021, except during a fire-restriction closure from 25 August to 29 September 2021. Visual encounter surveys were paired with a long-range PIT tag reader (Biomark HPR Plus and BP Lite antenna, Boise, Idaho, USA) to increase detection probability [84]. For captured individuals, we recorded dorsal body and substrate temperature (using an Extech® handheld infrared temperature gun), microhabitat (frog encountered in versus out of water), mass, SVL and Bd status. Capture locations were recorded using a Geode GNS3 GPS unit (Juniper Systems, Inc.) with sub-meter accuracy. Bd swabbing, extraction and qPCR procedures followed established protocols (supplementary material; [88,89]).

### 2.6. Statistical analysis

All statistical analyses were conducted in R Version 4.4.1 [90]. Packages *lme4* and *lmerTest* [91,92] were used to fit generalized linear mixed models (GLMMs) to characterize the effects of experimental treatment and individually selected temperature on response variables.

We used different subsets of temperature selection trial data to answer each of our research questions. To determine baseline selected temperature, we analysed 53 trials comprising all pre-treatment trials. For repeatability measures, we used trials from low treatment frogs that received two temperature selection trials (i.e. who experienced no changes) and the package *rptR* [93] to quantify repeatability of selected temperature and distance moved [45]. Repeatability is defined as the intraclass correlation coefficient (ICC), the proportion of total trait variation attributable to consistent differences among individuals, where values closer to 1 indicate higher consistency across repeated observations from the same individual. Repeatability estimates include two repeated observations per trialled individual. To quantify treatment effects on selected temperature, we analysed 98 trials for 49 individuals with both pre- and post-treatment trials. Models included treatment, timepoint (pre versus post), their interaction, and distance moved (to account for activity) as fixed effects. Frog identity and experimental replicate (one of six tanks, three high and three low treatment) were included as nested random effects. Activity levels in thermal behaviour trials (quantified as distance moved during the trial) may be correlated to selected temperature, so we included distance moved (in pixels) as a fixed effect in this model to account for nuisance variation [45]. We also used 126 post-treatment trials to analyse the effects of treatment and selected temperature on distance moved during trials, as well as effects of treatment and selected temperature on growth rate during the treatment period, with experimental replicate as a random effect. We included SVL at the time of the temperature trial as a fixed effect in models to account for variation in individual size.

For post-release analyses, we used 87 captures of 68 individual frogs to fit GLMMs to test for effects of treatment or selected temperature on five variables: canopy cover, air temperature, light intensity, frog microhabitat selection (in versus out of water) and relative frog body temperature (the frog's body temperature minus the ambient air temperature) at the place and time of capture (10 models). We also quantified the effects of treatment and selected temperature on frog dispersal, with dispersal distance quantified as the cumulatively summed Euclidean distances between each frog's subsequent detection locations, beginning at their release pool location (two models). For post-release recapture variables, we included data from all live frog detections during the post-release monitoring period (excluding dead bodies and loose encountered PIT tags). We included capture date, individual identity and release pool as random effects in all post-release habitat models. We did not have sufficient sample size to conduct formal statistical analysis for Bd results.

We used Cormack–Jolly–Seber (CJS) models to estimate weekly apparent survival and detection probability using the package *RMark* [94,95]. We considered models with the effects of treatment, selected temperature and body size (SVL) at release for both survival and detection. We used an information theoretical approach to compare support for 36 competing CJS models using Akaike's information criterion (AIC) method. We identified the model with the lowest AICc as the top-ranked model and considered models within approximately 2 AICc units to have similar support [96,97]. We considered a parameter uninformative if its 95% confidence interval (CI) for the β coefficient overlapped zero [98].

## 3. Results

### 3.1. Thermal treatment selection

In the *ex situ* tanks, high temperature frogs had access to maximum air temperatures of 30‒32°C on the basking platform, exceeding the critical thermal maximum for Bd but remaining within the range this species would experience in the wild (supplementary material; figure 1a). In the low temperature treatment, maximum available air temperatures ranged from 23°C to 25°C, consistent with standard husbandry protocols for this species. Frogs in both treatment groups also had access to cooler terrestrial microhabitats and chilled water, which remained stable at 15.9°C (s.e. = 0.001).

### 3.2. Repeatability of temperature selection trials

To assess repeatability of selected temperature and movement, we analysed data from 25 frogs in the low treatment group with two temperature selection trials each. Because these individuals did not experience a change in environmental conditions between trials, they provided a baseline estimate of individual consistency. Selected temperature in this group was significantly repeatable (ICC = 0.443, CI = 0.08–0.71, *p* = 0.010; supplementary material, figure S3). Distance moved during the trial was also significantly repeatable for these individuals (ICC = 0.550, CI = 0.23–0.75, *p* = 0.002; supplementary material, figure S4), indicating that both thermal selection and activity level are stable behavioural traits in recently metamorphosed *R. muscosa*.

### 3.3. Temperature selection and treatment effects on *R. muscosa*

Across all pre-treatment trials, selected temperature (IQR) of juveniles was 22.94–26.06°C (*n* = 53 trials; median = 24.54; mean ± s.e. = 24.53 ± 0.28°C). There was no difference in selected temperature between treatment groups in pre-treatment trials (figure 2). For high treatment frogs, selected temperature post-treatment decreased to 21.38–24.57°C (*n* = 63 trials; median = 23.05; mean ± s.e. = 23.15 ± 0.29°C). For low treatment frogs, the selected temperature post-treatment was very similar, at 23.28–26.69°C (*n* = 63 trials; median = 25.15; mean ± s.e. = 24.86 ± 0.29°C).

**Figure 2.**
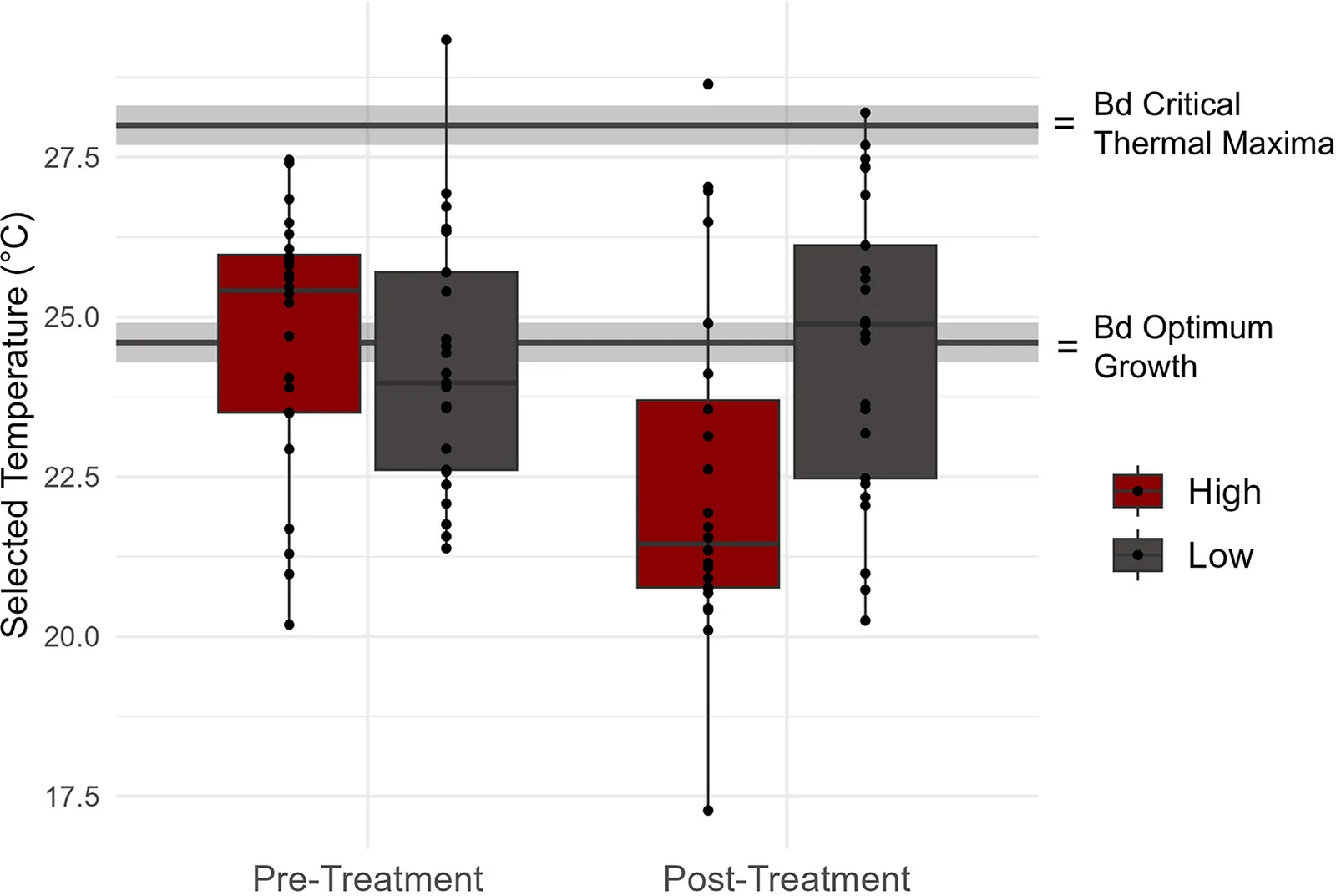
Selected temperatures of high and low treatment groups before and after exposure to thermal treatments.

After treatment, high temperature treatment frogs selected lower temperatures than those from the low temperature treatment. Among frogs tested both pre- and post-treatment (*n* = 49; *n* = 25 low and *n* = 24 high), high treatment frogs selected significantly lower temperatures after treatment than did low treatment frogs (figure 2; supplementary material, table S2.1). Mean selected temperature for high treatment frogs decreased by 2.22°C (s.e. = 0.66) whereas mean selected temperature for low treatment frogs increased by 0.25°C (s.e. = 0.46). Distance moved during a trial was negatively correlated with selected temperature (supplementary material, figure S5), indicating that more active frogs tended to select cooler regions in the arena.

### 3.4. Animal growth in laboratory

During the *ex situ* portion of the study, growth rate (SVL) did not differ between thermal treatments and was only marginally related to selected temperature (supplementary material, table S2.2; *β* = 0.03, *t*(121) = 1.77, *p* = 0.079). Thermal treatment had no detectable effect on growth rate (supplementary material, table S2.2; *β* = –0.08, *t*(4) = –0.65, *p* = 0.546). At release, SVL did not differ between high and low groups (*β* = –0.10, *t*(131) = –0.20, *p* = 0.841; supplementary material, table S2.3 and figure S6).

### 3.5. Bd outcomes

Of the 21 unique frogs hand-captured post-release, Bd swabs were collected from 16 individuals (high: *n* = 10; low: *n* = 6), with two individuals (both high treatment) being captured and swabbed twice. All swabs collected on the first recapture survey, 6 days after release, were Bd-negative (high: *n* = 5, low: *n* = 5), with the first positive Bd detection occurring 20 days after release on a high treatment frog (figure 3). Seven swabs from six frogs were positive for Bd, all from the high treatment group and all after the first recapture survey. No low treatment frogs were captured beyond one week post-release. Mean Bd infection intensity across seven positive swabs was 1 010 437 ITS copies (s.e. = 890 120, range = 898–6 340 591). One frog tested positive both on an early survey and again in October when it was recovered as a mortality. Bd-associated die-offs of frogs were also reported from a separate downstream release effort in the same watershed during the same year [88].

**Figure 3.**
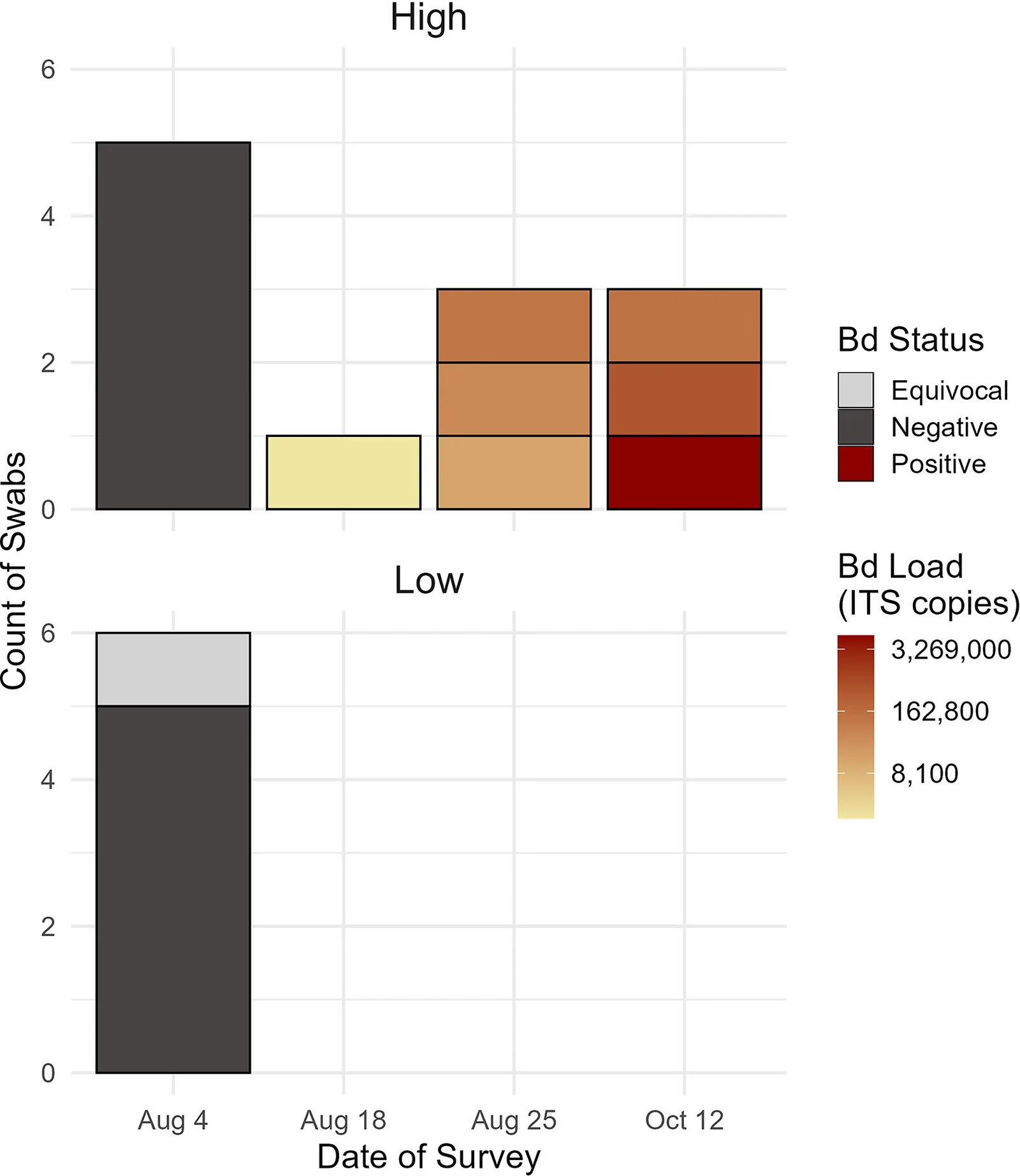
Bd status of recaptured frogs across post-release surveys for high (top) and low (bottom) treatment groups. No low-treatment Bd swabs were collected after 4 August. Non-grey bars represent positive Bd swabs, with warmer colours indicating high Bd load (ITS = internal transcribed spacer).

### 3.6. Environmental variables

Because most detections occurred within the first month post-release, when frogs are dispersing and may not yet be selecting fine-scale microhabitats, our ability to detect differences in habitat use was limited. Frogs did not show evidence of differential microhabitat selection in the field, regardless of their selected temperature or treatment groups for any of the post-release environmental variables we measured (canopy cover, air temperature, microhabitat selection and light intensity; supplementary material, tables S3.1 and S3.2). Similarly, cumulative dispersal distance from the release pool (supplementary material, table S3.3) did not differ among treatment groups and was not associated with individual selected temperatures. Relative body temperatures (the frog's body temperature minus the ambient air temperature) collected from 27 frogs in the field (high: *n* = 17, low: *n* = 10) were widely variable and were similar for high treatment frogs (mean = –15.1°C, s.e. = 3.1, range = –37.3–1.1°C) and low treatment frogs (mean = –20.8°C, s.e. = 3.2, range = –35.3–5.8°C; supplementary material, figure S7, tables S3.1 and S3.2), with frogs from both treatment groups nearly always being colder than ambient air temperatures. Raw frog body temperatures recorded in the field for both treatment groups (high: mean = 17.9, s.e. = 1.5, range = 0.7–24.2°C; low: mean = 18.8, s.e. = 1.3, range = 13.1–28.0°C) were consistently lower than temperatures selected during the pre-treatment lab trial (IQR = 22.94–26.06°C; median = 24.54; mean ± s.e. = 24.53 ± 0.28°C).

### 3.7. Mark recapture results

During the four months following release, 70 of the 124 PIT-tagged frogs were detected at least once (98 unique detections). Most live detections (84 of 87, 96.5%) occurred within the first month, with very few detections thereafter (supplementary material, figure S8). Nine detections were mortalities (high: *n* = 5; low: *n* = 4) and two were loose PIT tags. The lowest-AICc CJS model included both treatment and SVL at release for both apparent survival and detection probability, although several models received similar support (supplementary material, table S4.1). Based on the lowest AICc model, high treatment frogs had higher weekly apparent survival (77.5%, CI: 68.1%–84.8%) than low treatment frogs (63.3%, CI: 53.0%–72.6%) in the 4 months of surveys following release; larger individuals had higher apparent survival; and detection probability was higher for low treatment frogs and for smaller frogs (figure 4a,b; supplementary material, tables S4.2 and S4.3). No live frogs were detected beyond 11 weeks post-release during the post-release monitoring for this study; more than 15 additional surveys at this site from 2022 to 2025 have not detected any individuals from the 2021 release cohort, suggesting treatment did not have meaningful impacts on longer-term outcomes.

**Figure 4.**
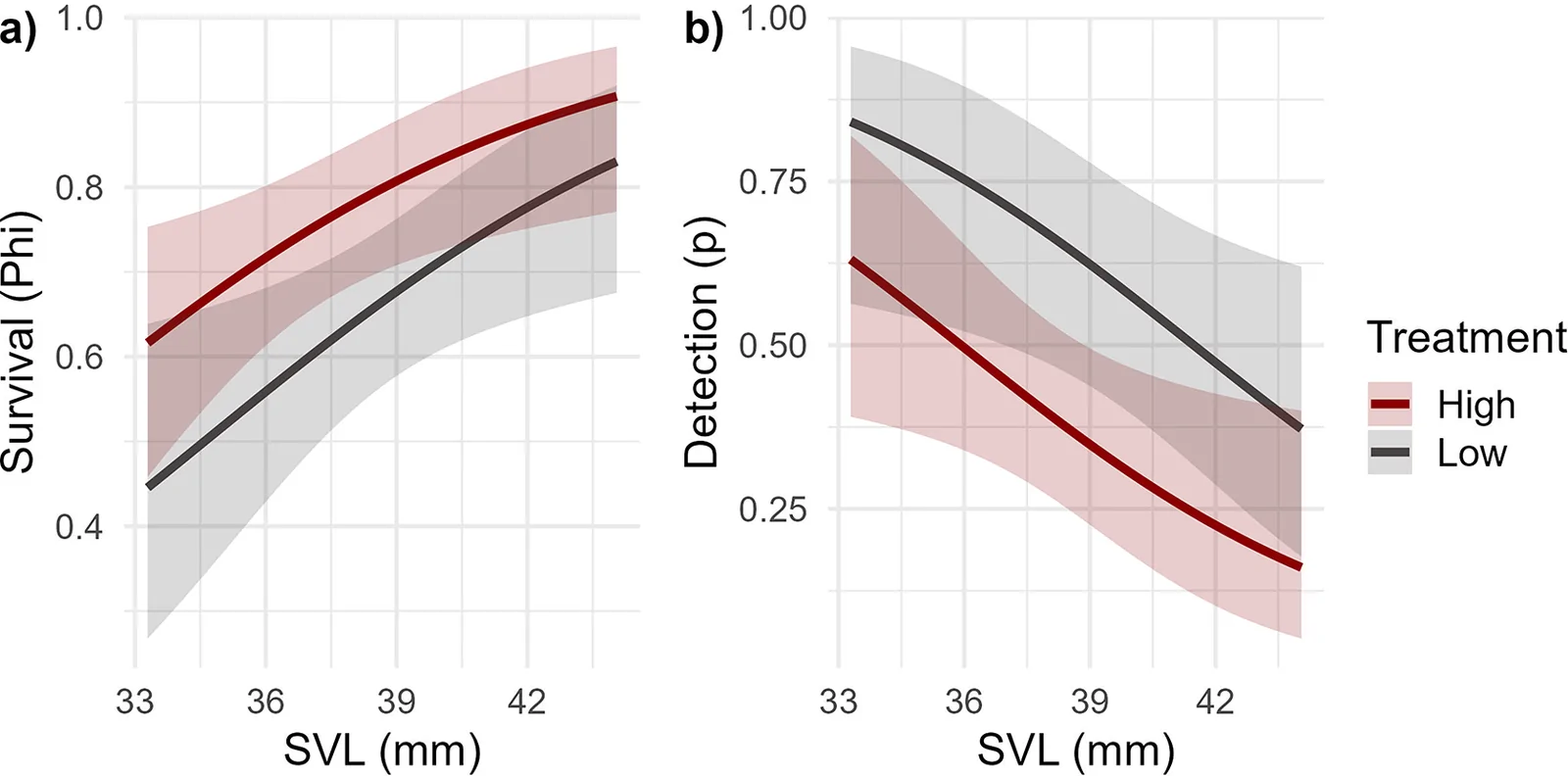
Modelled weekly apparent survival (a) and detection probability (b) of released frogs for high and low treatment groups as a function of snout-vent-length (SVL) at release.

## 4. Discussion

Understanding how early-life environments shape thermal physiology is critical for amphibians threatened by temperature-sensitive pathogens, such as Bd. This is particularly true for temperate, semi-aquatic species like *R. muscosa* occupying cool, high-elevation sites where cool water may make it difficult to clear infections. Here, we tested whether *ex situ* thermal conditions during development altered selected temperatures and influenced post-release microhabitat use, disease outcomes or early survival. These questions are essential because reintroductions of Bd-susceptible amphibians frequently fail shortly after release, and effective husbandry interventions are urgently needed. We found that thermal treatment affected selected temperatures in *R. muscosa*, though contrary to our predictions, frogs exposed to high basking temperatures selected cooler temperatures in standardized thermal arenas. Although these individuals exhibited marginally higher short-term apparent survival, we found no evidence that thermal acclimation improved establishment following release. Together, these findings underscore the value of understanding the intricate link between *ex situ* husbandry conditions and survival outcomes in the wild when rearing animals for reintroductions, particularly for species threatened by thermally sensitive pathogens.

### 4.1. Selected temperature in *R. muscosa*

Consistent with findings for other amphibian species, our results show that selected temperature for this population of *R. muscosa* varied by individual and is repeatable for individuals over time [40,45]. Our trials focused on recently metamorphosed frogs, a life stage particularly vulnerable to Bd infection and mortality in this species [27]. The selected temperature range (IQR) of 22.94–26.06°C and mean ± s.e. selected temperatures of 24.53 ± 0.3°C for recently metamorphosed frogs aligns with the reported lab mean selected daytime temperature of 24.3 ± 0.6°C (± s.e.) for adult *Rana sierrae*, the sister species to *R. muscosa* [79]. While the genotypes and phenotypes of Bd present in southern California have not been thoroughly described, the CJB5-2 isolate of Bd from northern California grows successfully between 2°C and 25°C, reaching optimum growth at 24.60°C and a critical maximum near 27–28°C [29]. Of all temperature trials conducted in our study, only five frogs in six trials (2.8% of frogs) selected temperatures warm enough to exceed Bd's thermal maxima. Characterization of the isolate of Bd that occurs in the study region and the thermal limits of that isolate are important next steps in understanding these dynamics. The proximity of the temperatures selected by *R. muscosa* to the optimal growth range for Bd could limit opportunities for behavioural avoidance of infection. Although many ranid frogs select temperatures conducive to Bd growth, *R. muscosa* occupies cold, high-elevation habitats that may restrict access to warmer microhabitats capable of suppressing Bd, potentially exacerbating disease impacts.

We conducted temperature selection trials in the warm spring and summer active season, and frogs spent little time in cold portions of the arena (474 frames across all trials, 1.09% of frames where frogs selected temperatures less than 15°C). Temperature selection is seasonally plastic in amphibians, and temperatures selected during active season trials may not reflect temperature requirements at other times of year [56,99,100]. Also, our temperature treatment window was relatively short (approx. three months) compared with the total *ex situ* rearing time frame for this species, so it is possible that the observed shifts in selected temperature were more aligned with short-term acclimation. Nonetheless, even short-term shifts in temperature selection that align with periods of high disease risk for an organism (immediately post-release, overwintering) may still be beneficial. Thermal microhabitat selection during active periods can reduce Bd infection, but in highly seasonal environments with long winter periods, amphibians are constrained by environmental thermal heterogeneity. For aquatic over-winterers like *R. muscosa* and *R. sierrae*, Bd may be particularly impactful during this period when the pathogen can outcompete host immune function [101–103].

Surprisingly, high temperature treatment frogs selected lower temperatures than low treatment frogs. This result could reflect several non-mutually exclusive mechanisms. It may represent a shift in thermal preference, which could be due to a learned aversion to warmer substrates if the highest available temperatures exceeded thermal tolerance, or a transient stress response that may dissipate over time. We anecdotally observed frogs in the high treatment moving away from the hottest part of the basking platform in their treatment tanks, while individuals in the low treatment were observed using the entire basking surface. Thermal refugia were available in both aquatic and terrestrial microhabitats for both groups of frogs and water temperatures remained stable (approx. 16°C) across all replicates, but the hottest available habitat in high treatment replicates may have exceeded voluntary thermal maxima, causing frogs to seek cooler microhabitats. FLIR thermal cameras were used to measure basking platform surface temperature (rather than air temperature approx. 1 cm above platforms as recorded by temperature loggers), and surface temperatures in the high treatment group reached as high as 47°C. Although there were lower temperatures available on the basking platforms and in the water in both treatments, the reduction in selected temperature by high temperature treatment frogs could be an antagonistic response to thermal stress (i.e. heat hardening). Because we manipulated thermal opportunity rather than realized exposure and did not quantify body temperatures during the treatment period, we cannot determine the exact temperatures experienced by individuals. Given the availability of overlapping microhabitats across treatments, realized thermal differences were likely smaller than the differences in maximum temperatures. This may help explain the counterintuitive shift in selected temperature and future work could quantify realized exposure and manipulate thermal environments more extensively throughout development to study the impacts on thermal limits and preference [45].

### 4.2. Outcomes in the field

The lowest-AICc model suggested that frogs exposed to higher temperatures had higher apparent survival and were detected less frequently than frogs in the low treatment, although support for treatment effects was limited. Given sparse detections, particularly beyond the first few weeks post-release, and substantial model uncertainty, these results should be interpreted cautiously. Overall survival was low, with detections declining rapidly and no individuals from either treatment group persisting beyond the initial post-release period. Larger juveniles exhibited higher short-term apparent survival, a pattern consistent with previous work in anuran species [104]. We were unable to draw robust conclusions about Bd dynamics due to limited and uneven sampling across time and treatment groups. Taken together, the available data provide only weak evidence for treatment effects on short-term survival and do not indicate establishment under either thermal regime.

There were no differences between treatment groups and no correlations between selected temperature and any of the microhabitat variables we measured. Most of our detections were in the month after release when frogs are dispersing and are unlikely to be prioritizing selecting microhabitat, so it is unsurprising that there was no consistent difference between treatment groups for the habitat variables we tested. Interestingly, frog body temperatures collected in the field for both treatment groups were lower than temperatures selected during pre-treatment lab trials, similar to results reported for *R. sierrae* [79]. This suggests that available conditions at this field site may not provide adequate opportunity for animals to reach their selected temperature ranges.

That temperature selection may carry over from rearing conditions to post-release environments is intriguing and provides a potential avenue of future research if it can be paired with short-term Bd mitigation efforts, such as controlled infection and clearing before release (i.e. immune priming, which could reduce Bd susceptibility on future exposure). Implementing these methods could facilitate increased survival through the post-release dispersal period, providing more data on microhabitat selection that may better inform the choice of future release locations. Indeed, increased survival of juvenile life stages is critical for population establishment and growth in amphibian taxa [104]. The mechanism underlying the possible association between temperature treatment and short-term apparent survival remains unclear. It is possible that the broader thermal conditions in high treatment tanks (figure 1a) prepared them for variable field conditions by encouraging shuttling around the tank to find optimum thermal environments. Also, detection rates were higher for low treatment frogs than for high treatment frogs, despite there being no difference in body size when they were released, which may reflect differences in habitat use, although data were limited to evaluate this directly. Given the limited number of recaptures and model uncertainty, these survival and detection results should be interpreted as exploratory.

### 4.3. Conservation recommendations

Thermal conditioning changed selected temperatures and coincided with slightly higher short-term apparent survival but did not result in frog establishment at a Bd-positive site. Multiple reintroduction attempts at this location have resulted in limited long-term survival, emphasizing the severity of Bd impacts in this region [89]. Given that population estimates for *R. muscosa* remain extremely low throughout southern California [96], the development of effective pre-release interventions is a pressing conservation need. The imperilled status of this species poses a challenge to conducting studies with adequate sample size. The 134 animals available for this study were from the same clutch and were therefore lacking genetic variation. While our results can only be interpreted in the context of one parentage of *R. muscosa*, it is noteworthy that there is limited extant genetic diversity in this species, and a genetic management plan is critical for future conservation planning.

Our findings demonstrate that temperature selection for *R. muscosa* is plastic and can be shifted in the short-term via naturalistic husbandry conditions providing a range of thermal microhabitats. Husbandry and rearing conditions can have unexpected but critical impacts on survival in the wild; thus, conditions in *ex situ* facilities should be carefully trialled and tested to maximize reintroduction success. Naturalistic, variable and cycling temperature treatments that integrate opportunities for both high temperature basking and cool temperature refugia could be important for amphibian rearing programmes. Temperature during rearing of other life stages (e.g. tadpoles) could impact selected temperature as well and is a potential avenue of future research [45]. However, when releasing Bd-susceptible amphibians into Bd positive sites, our findings suggest that such efforts must be paired with explicit Bd mitigation tools. Bd-related declines remain a significant challenge, but emerging conservation tools—such as immunizations, behavioural conditioning and habitat alteration—offer promising future directions [105]. Such interventions could be tested in conjunction with pre-release husbandry modifications to optimize outcomes in Bd positive environments. Future work integrating thermal conditioning with targeted Bd management, particularly during the vulnerable post-release dispersal and overwintering periods, may help improve survival beyond the first month following release. Experimental combinations of husbandry modifications and disease interventions represent a promising direction for enhancing reintroduction outcomes for Bd-susceptible amphibians.

## Supplementary Material



## Data Availability

Data and code are available via Figshare [106]. Supplementary material is available online [107].
