# Supplementary material for: Ex situ thermal acclimation alters temperature selection with limited post-release effects in an endangered frog (Rana muscosa)
Source: R Soc Open Sci. 2026 Oct 7;13(10):260332. doi: 10.1098/rsos.260332 (PMC13641914; doi:10.1098/rsos.260332)
Supplement: Supplementary file 1 [file rsos.260332.zip › rsos.260332/rsos260332_si_001.docx]

**Supplementary Materials**

**Title:** Ex situ thermal acclimation during development affects temperature selection but not reintroduction performance in an endangered frog (*Rana muscosa*)

**Authors:** Kenzi M. Stemp^1,3^, Talisin T. Hammond^1^, Michel E. B. Ohmer^2^, Gregory F. Grether^3^, and Debra M. Shier^1,3^

**Affiliations:**

1. San Diego Zoo Wildlife Alliance, 15600 San Pasqual Valley Rd. Escondido, CA 92027, USA

2. Dept. of Biology, University of Mississippi, University, MS, 38677, USA

3. University of California, Los Angeles, 621 Charles E. Young, Dr. South, Los Angeles, CA 90094, USA

*Husbandry Conditions for ex situ RAMU Facility*

Experimental tanks were 284 L, 122 x 51 x 46 cm glass aquaria with tight fitting metal mesh lids and were outfitted with a filter (Fluval 407 Canister), water chiller (JB Arctica 1/4HP), and UVB (Reptisun T8 24-inch UVB bulbs) and basking lights (Zilla Mini Hallogen dome, 50W). To mimic natural light conditions, daily duration of both UVB lights and basking lights was increased in three stages through the length of the experiment to mimic progression of natural seasonal light availability.

Tanks included environmental enrichment in the form of natural backgrounds and substrate, flowing water, gravel and rock perches, two plastic plants, and variable water depth availability (26cm - 1cm). Water temperature and pH were monitored daily and other water quality parameters (ammonia, nitrate, nitrite, chlorine, TDS, phosphorous, iron, and copper) were monitored weekly, bi-weekly, or monthly. Animals were fed a varied diet including size-appropriate bean beetles and flightless fruit flies (produced at UCLA), and blackfly larvae and small crickets (Rainbow Mealworms) for metamorphosed froglets, and Soilent Green and Bottom Scraper Gels (Repashy) and algae wafers (Hikari) for tadpoles. Animals were received at the UCLA facility as tadpoles in October of 2020 and were held at water temperatures between 15-16°C through the fall until the brumation period began. To mimic natural overwintering conditions, we brumated all individuals from 20 January 2021 to 6 April 2021 (from the start of the cooling period to the end of the warming period). To enter and exit the brumation period, water temperatures in tanks were raised or lowered at a rate of 2°C every 2 days for 18 days and were held at 15-16°C once the entering/exit phase was complete. Metamorphosis for this clutch varied with individuals completing metamorphosis between 13 April - 10 June 2021.

*Thermal treatment range testing*

Basking light availability and intensity regimes were selected using data from 1) replica 3% agar frog models, 2) reported known physiological temperature ranges for the species, and 3) knowledge of the range of temperatures available in local habitats [1].

1) Replica 3% agar frogs implanted with iButton temperature loggers (DS1921G-F5 Thermochron, 4K Datalogger) [2–4] were paired with a temperature and light intensity datalogger (HOBO Pendant Temperature/Light 64K Datalogger) and distributed across a range of light and substrate conditions at a field site in March of 2021. Agar frog models recorded temperatures from 7.5-21°C (mean=12.24, SD=4.11) in March.
2) Although the Bd genotypes present in southern California have not yet been described, Bd isolated from *Rana sierrae* (the Sierra Nevada yellow-legged frog, hereafter referred to as RASI) had a reported thermal range of 2‒25°C with maximum growth at 24.6°C and T_max_ ~27-28°C [5].
3) Six HOBO dataloggers were deployed at a local field site from 26 February 2021 - 23 June 2021; two HOBOs were deployed in water (at 1cm and 40cm depths) and four HOBOs were terrestrial in low, moderate, and high light conditions. Between February-June of 2021, air temperatures recorded by HOBO loggers at the same site were 4.21‒44.93°C (mean=15.77, SD=9.01), and water temperatures were 6.98-30.14°C (mean=12.90, SD=4.29).

*Thermal treatment implementation*

To create basking treatment conditions in the lab, heat lamps (Zilla Mini Hallogen Dome) with 50W bulbs were positioned 50cm above basking platforms and an automatic light timer controlled daily duration to mimic outdoor conditions. For “High” treatments we used two lights to increase temperature and light intensity, with both lights focused on same point. Temperatures in tanks were monitored with Onset HOBO®, temperature and light loggers (one on basking platform under basking light and one underwater) and one iButton® (DS1921G-F5# Thermochron, 4K) (mounted to side of tank). We used the same replica 3% agar frogs to test lamp positioning and the conditions a frog would experience in experimental tanks before implementing the treatment. We also opportunistically used the FLIR thermal camera to measure surface temperatures on basking platforms once for each tank in each “seasonal” temperature regime; the goal of these measurements were to understand the maximum temperature on the actual surface of the basking platform (rather than ~1 cm above the basking platform, which is what the HOBO® temperature loggers measure).

*Inter-observer agreement for thermal preference trial scoring*

Before scoring trial files, all scorers first scored a calibration file during training (Fig. S6) to test inter-observer agreement (function rpt() in package rptR, Version 0.9.22). Repeatability scores between observers for temperature and coordinate values were high for temperature (R=0.945, SE=0.006, CI=[0.932, 0.956]), x coordinates (R=0.900, SE=0.011, CI=[0.876, 0.917]) and y coordinates (R=0.903, SE=0.011, CI=[0.876, 0.921]).

*Temperature selection trial scoring methodology*

Trials were manually scored using FLIR Research Studio software (Teledyne FLIR, Version 2.1). To score a file, scorers manually input environmental parameters into FLIR Research Studio to calibrate the thermal file for local conditions (distance from object, room temperature, and relative humidity). Then, scorers collected the frame number, (x,y) coordinate of the frog in each frame, and temperature of frog in each frame (frames/trial: N=236.16, SD=0.65). Files were given a unique name with trial metadata, and all scored files were summarized with a custom function in R Studio.

*Bd swabbing methodology and swab processing*

All frogs were swabbed using a standard protocol (<https://science.sandiegozoo.org/sites/default/files/sites/default/files/blogimages/Chytrid%20Guidelines%202021.pdf>). Swabs were processed by SDZWA’s Amphibian Disease laboratory. DNA was extracted using Prepman Ultra (Applied Biosystems) and  amplified using a quantitative Polymerase Chain Reaction (qPCR) assay [6,7] that used a synthetic plasmid-based standard (Pisces Molecular LLC, Boulder, CO. B. dendrobatidis, B. salamandrivorans, Ranavirus plasmid qPCR Control Kit) [8–11].The plasmid contains a target sequence for Bd’s ITS-1 rDNA gene; this method quantifies the number of ITS-1 copies per sample [12]. Samples were run in triplicate. Further details can be found in Hammond et al., 2025 and 2021.

**Figure S1.** Temperature and light conditions recorded by HOBO temperature logger on the basking platform in high (circles) and low (squares) treatment tanks.

**Figure S2.** Timeline of project events from temperature selection trials through field surveys

**Figure S3.** Interobserver agreement in scoring data. Plots of frog travel paths recorded for the calibration temperature preference trial to validate inter-observer agreement. Each panel represents the path of the same individual frog and trial, scored by a different scorer – note the high level of similarity between observers.

**Figure S4.** Repeatability trend in selected temperature for frogs in the low treatment group that received two temperature preference trials. Selected temperature in a frog’s first trial are on the x-axis and selected temperature in the same frog’s second trial are on the y-axis. Only low treatment frogs were tested for repeatability because they did not experience a change in conditions that would affect their selected temperatures.

**Figure S5.** Repeatability trend in distance moved during a trial for frogs in the low treatment group that received two temperature preference trials. Distance moved (in pixels) in a frog’s first trial are on the x-axis and distance moved in the same frog’s second trial are on the y-axis. Only low treatment frogs were tested for repeatability because they did not experience a change in conditions that would affect their selected temperatures.

**Figure S6.** Relationship between distance moved during a trial (in pixels) and preferred temperature for both treatment groups.

**Figure S7.** Snout-vent-length (SVL) at monthly measurements for metamorphosed frogs held in high treatment (grey) and low treatment (red) conditions. Points represent individual frogs.

**Figure S8**. Relative body temperatures calculated as the difference between frog body temperature collected with an infrared thermometer and ambient air temperature at the time of capture during field surveys. Points represent individual frogs.

**Figure S9.** Count of post-release frog detections during each survey date following release into the field site. Grey bars represent detections of high treatment frogs and red bars represent detections of low treatment frogs.

1. **Treatment Conditions**

Table S1. Target maximum temperatures, number of basking and UV lights available in experimental tanks, and hours of basking light available in season stages for experimental treatments. Minimum temperatures in both treatments were similar at 16–17°C. “Spring” conditions lasted from May 10 2021 to June 1 2021, “Late Spring” conditions lasted from June 1 2021 to June 28 2021, and “Summer” conditions lasted from June 28 2021 to release on July 29 2021.

| Treatment | Target Max Temp. (°C) | N Basking Lights | Hours Light “Spring” | Hours Light “Late Spring” | Hours Light  “Summer” | N Basking Platforms | N UV Lights |
| --- | --- | --- | --- | --- | --- | --- | --- |
| *High* | 29–30 | 2 | 4 | 6 | 8 | 1 | 1 |
| *Low* | 22–25 | 1 | 2 | 3 | 4 | 1 | 1 |

2. **Lab results**

**Table S2.1** Estimates from GLMM models for the effects of experimental treatment, trial timepoint (pre- or post-treatment), distance moved, and snout-vent-length (SVL) at the time of the trial on selected temperature (in °C). The model includes individual identity and experimental replicate (one of six, three high and three low experimental tanks) as random effects. Distance moved and trial SVL were scaled to coerce predictors to similar scales. Data includes 98 trials for 49 frogs that were trialed twice (High: N=24 frogs, Low: N=25 frogs)

|  | Estimate | Standard Error | Degrees of Freedom | t value | p value |
| --- | --- | --- | --- | --- | --- |
| *(Intercept)* | 24.1603 | 0.5146 | 19.0863 | 46.953 | <0.001 |
| *Treatment (low)* | -0.3013 | 0.6029 | 10.5798 | -0.500 | 0.627 |
| *PrePost (post)* | -1.1200 | 0.8163 | 89.8816 | -1.372 | 0.173 |
| *Distance Moved* | -1.0812 | 0.2171 | 80.2675 | -4.980 | <0.001 |
| *SVL* | -0.3321 | 0.3654 | 73.5437 | -0.909 | 0.366 |
| *Treatment (low)**  *PrePost (post)* | 2.1518 | 0.7459 | 47.2323 | 2.885 | 0.005 |

**Table S2.2** Estimates from GLMM models for the effects of experimental treatment, selected temperature, and SVL at the time of the trial on growth rate (SVL, mm/month) for thermal preference trials. The model includes experimental replicate (one of six, three high and three low experimental tanks) as a random effect. Data include 126 trials for 126 frogs (High: N=63 frogs, Low: N=63 frogs).

|  | Estimate | Standard Error | Degrees of Freedom | t value | p value |
| --- | --- | --- | --- | --- | --- |
| *(Intercept)* | 0.87837 | 0.46685 | 120.4196 | 1.881 | 0.062 |
| *Treatment (low)* | -0.08296 | 0.12716 | 4.4517 | -0.652 | 0.546 |
| *Selected temperature* | 0.03513 | 0.01983 | 121.3260 | 1.771 | 0.079 |
| *SVL* | 0.26935 | 0.04495 | 118.8387 | 5.993 | <0.001 |

**Table S2.3** Estimates from GLMM models for the effects of experimental treatment on frog size at the time of release (SVL). The model includes experimental replicate (one of six, three high and three low experimental tanks) as a random effect. Data include frog size measurements for 134 individuals at the time of release.

|  | Estimate | Standard Error | Degrees of Freedom | t value | p value |
| --- | --- | --- | --- | --- | --- |
| *(Intercept)* | 37.5318 | 0.3571 | 131 | 105.096 | <0.001 |
| *Treatment (low)* | -0.1014 | 0.5032 | 131 | -0.201 | 0.841 |

3. **Field Study Results**
   3.1 **Effects of Treatment on Habitat Variables**

Table S3.1 Estimates from five separate GLMM models for the effects of experimental treatment on habitat variables at recapture location for post-release frog captures; for all GLMMs, the base level of the “treatment” factor was “high.” Air temperature and light intensity reflect an average of measurements from 30 minutes prior to and following a frog’s capture. All models include date of survey (5 dates), PIT tag (i.e. individual identity), and release pool (5 assigned release locations) as random effects. Data include 87 capture instances for 68 unique frogs.

| Variable | Unit | Estimate | Standard Error | Degrees of Freedom | | t (z) value | p value |
| --- | --- | --- | --- | --- | --- | --- | --- |
| *Canopy gap area* | pixels | -48.376 | 17750 | 63.140 | -0.003 | | 0.998 |
| *Air temperature* | °C | 0.9433 | 2.4818 | 80.284 | 0.380 | | 0.705 |
| *Light*  *intensity* | lux | 525.555 | 983.901 | 82.817 | 0.534 | | 0.595 |
| *Relative frog body temperature* | °C | -5.131 | 4.790 | 24.767 | -1.071 | | 0.294 |
| *Microhabitat* | binary, in (0) or out (1) of water | 0.541 | 0.542 | - | (0.998) | | 0.318 |

**3.2 Effects of Selected Temperature on Habitat Variables**

Table S3.2 Estimates from five separate GLMM models for the effects of selected temperature on habitat variables at recapture location for post-release frog captures. Air temperature and light intensity reflect an average of measurements from 30 minutes prior to and following a frog’s capture. All models include date of survey (5 dates), PIT tag (i.e. individual identity), and release pool (5 assigned release locations) as random effects. Data include 86 capture instances for 67 unique frogs.

| Variable | Unit | Estimate | Standard Error | Degrees of Freedom | | t (z) value | p value |
| --- | --- | --- | --- | --- | --- | --- | --- |
| *Canopy gap area* | pixels | 641.490 | 3651.530 | 65.35 | 0.176 | | 0.861 |
| *Air temperature* | °C | -0.113 | 0.505 | 77.776 | -0.224 | | 0.823 |
| *Light*  *intensity* | lux | -37.010 | 201.320 | 79.330 | -0.184 | | 0.855 |
| *Relative frog body temperature* | °C | -1.981 | 1.069 | 23.751 | -1.854 | | 0.076 |
| *Microhabitat* | binary, in (0) or out (1) of water | 0.229 | 0.124 | - | (1.841) | | 0.066 |

3.3 **Euclidean distance traveled from release pool**

Table S3.3 Estimates from GLMM models for the effects of experimental treatment and selected temperature on dispersal distance (in meters) for post-release frog captures. All models include date of survey (5 dates), PIT tag (i.e. individual identity), and release pool (5 assigned release locations) as random effects. Data include 85 capture instances for 65 unique frogs.

| Predictor | Estimate | Standard Error | Degrees of Freedom | | t value | p value |
| --- | --- | --- | --- | --- | --- | --- |
| *Treatment* | 6.016 | 12.170 | 56.690 | 0.494 | | 0.623 |
| *Selected temperature* | -1.112 | 2.490 | 59.840 | -0.447 | | 0.657 |

4. **RMark Resutls**

Table S4.1 Comparison of Cormack-Jolly-Seber models for Phi (survival) and p (detection probability) for all candidate models. Models are ranked by ΔAICc and explanatory variables include treatment, preferred temperature, and SVL at release.

|  | Model | Par | AIC | ΔAIC | Weight | Deviance |
| --- | --- | --- | --- | --- | --- | --- |
| 36 | Phi(~Treatment + SVL) p(~Treatment + SVL) | 6 | 395.606 | 0 | 0.184625 | 383.6063 |
| 8 | Phi(~SVL) p(~SVL) | 4 | 397.832 | 2.2261 | 0.060658 | 389.8325 |
| 12 | Phi(~SVL) p(~Treatment + SVL) | 5 | 398.026 | 2.4205 | 0.055041 | 388.0268 |
| 7 | Phi(~SVL) p(~1) | 3 | 398.067 | 2.4609 | 0.05394 | 392.0672 |
| 1 | Phi(~1) p(~1) | 2 | 398.150 | 2.5442 | 0.051739 | 31.92796 |
| 20 | Phi(~Tpref + SVL) p(~SVL) | 5 | 398.732 | 3.1260 | 0.038679 | 388.7324 |
| 32 | Phi(~Treatment + SVL) p(~SVL) | 5 | 398.760 | 3.1539 | 0.038144 | 388.7602 |
| 35 | Phi(~Treatment + SVL) p(~Treatment) | 5 | 398.889 | 3.2832 | 0.035755 | 388.8896 |
| 19 | Phi(~Tpref + SVL) p(~1) | 4 | 399.167 | 3.5612 | 0.031116 | 391.1675 |
| 31 | Phi(~Treatment + SVL) p(~1) | 4 | 399.220 | 3.6145 | 0.030298 | 391.2208 |
| 11 | Phi(~SVL) p(~Treatment) | 4 | 399.368 | 3.7624 | 0.028137 | 391.3688 |
| 24 | Phi(~Tpref + SVL) p(~Treatment + SVL) | 6 | 399.389 | 3.7833 | 0.027845 | 387.3897 |
| 5 | Phi(~1) p(~Treatment) | 3 | 399.407 | 3.8013 | 0.027595 | 31.18511 |
| 13 | Phi(~Tpref) p(~1) | 3 | 399.602 | 3.9965 | 0.02503 | 393.6029 |
| 25 | Phi(~Treatment) p(~1) | 3 | 399.736 | 4.1304 | 0.023409 | 31.51416 |
| 10 | Phi(~SVL) p(~Tpref + SVL) | 5 | 399.793 | 4.1871 | 0.022754 | 389.7935 |
| 2 | Phi(~1) p(~SVL) | 3 | 399.807 | 4.2008 | 0.022598 | 393.8072 |
| 29 | Phi(~Treatment) p(~Treatment) | 4 | 399.807 | 4.2015 | 0.022592 | 29.58522 |
| 9 | Phi(~SVL) p(~Tpref) | 4 | 399.819 | 4.2131 | 0.022461 | 391.8194 |
| 3 | Phi(~1) p(~Tpref) | 3 | 399.916 | 4.3097 | 0.021401 | 393.9161 |
| 22 | Phi(~Tpref + SVL) p(~Tpref + SVL) | 6 | 400.559 | 4.9527 | 0.015517 | 388.5591 |
| 34 | Phi(~Treatment + SVL) p(~Tpref + SVL) | 6 | 400.592 | 4.9857 | 0.015263 | 388.5921 |
| 6 | Phi(~1) p(~Treatment + SVL) | 4 | 400.656 | 5.0502 | 0.014779 | 392.6566 |
| 30 | Phi(~Treatment) p(~Treatment + SVL) | 5 | 400.706 | 5.1003 | 0.014413 | 390.7067 |
| 33 | Phi(~Treatment + SVL) p(~Tpref) | 5 | 400.715 | 5.1087 | 0.014353 | 390.7150 |
| 23 | Phi(~Tpref + SVL) p(~Treatment) | 5 | 400.750 | 5.1436 | 0.014104 | 390.7500 |
| 17 | Phi(~Tpref) p(~Treatment) | 4 | 401.087 | 5.4808 | 0.011916 | 393.0871 |
| 21 | Phi(~Tpref + SVL) p(~Tpref) | 5 | 401.166 | 5.5606 | 0.01145 | 391.1669 |
| 14 | Phi(~Tpref) p(~SVL) | 4 | 401.261 | 5.6552 | 0.010921 | 393.2616 |
| 27 | Phi(~Treatment) p(~Tpref) | 4 | 401.331 | 5.7246 | 0.010549 | 393.3310 |
| 26 | Phi(~Treatment) p(~SVL) | 4 | 401.405 | 5.7991 | 0.010163 | 393.4054 |
| 15 | Phi(~Tpref) p(~Tpref) | 4 | 401.584 | 5.9782 | 0.009292 | 393.5846 |
| 4 | Phi(~1) p(~Tpref + SVL) | 4 | 401.662 | 6.0558 | 0.008939 | 393.6622 |
| 18 | Phi(~Tpref) p(~Treatment + SVL) | 5 | 402.394 | 6.7883 | 0.006198 | 392.3947 |
| 28 | Phi(~Treatment) p(~Tpref + SVL) | 5 | 403.121 | 7.5151 | 0.004309 | 393.1214 |
| 16 | Phi(~Tpref) p(~Tpref + SVL) | 5 | 403.261 | 7.6551 | 0.004018 | 393.2614 |

Table S4.2. Beta Estimates for the best fit Cormack-Jolly-Seber model, Phi(~Treatment + SVL) p(~Treatment + SVL). SVL indicates frog SVL at the time of release and estimates explain weekly apparent survival.

|  | Estimate | Standard  Error | Lower CI | Upper CI |
| --- | --- | --- | --- | --- |
| Phi*(Intercept) | -5.11409 | 2.468848 | -9.95304 | -0.27515 |
| Phi*Treatment | -0.69245 | 0.335937 | -1.35089 | -0.03402 |
| Phi*SVL | 0.167853 | 0.066938 | 0.036654 | 0.299053 |
| p*(Intercept) | 7.310675 | 3.43718 | 0.573803 | 14.04755 |
| p*Treatment | 1.130312 | 0.512236 | 0.12633 | 2.134295 |
| p*SVL | -0.20351 | 0.090626 | -0.38114 | -0.02588 |

Table S4.3. Real Estimates for the best fit Cormack-Jolly-Seber model, Phi(~Treatment + SVL) p(~Treatment + SVL). SVL indicates frog SVL at the time of release and estimates explain weekly apparent survival.

|  | Estimate | Standard Error | Lower CI | Upper CI |
| --- | --- | --- | --- | --- |
| Phi High | 0.775384 | 0.042824 | 0.68071 | 0.848244 |
| Phi Low | 0.633326 | 0.050484 | 0.53007 | 0.725637 |
| p High | 0.403249 | 0.069664 | 0.27701 | 0.543755 |
| p Low | 0.676639 | 0.092850 | 0.47667 | 0.827801 |

References

1.	Knapp RA, Briggs CJ, Smith TC, Maurer JR. 2011 Nowhere to hide: impact of a temperature-sensitive amphibian pathogen along an elevation gradient in the temperate zone. *Ecosphere* **2**. (doi:10.1890/es11-00028.1)

2.	Roznik EA, Alford RA. 2014 Using pairs of physiological models to estimate temporal variation in amphibian body temperature. *J Therm Biol* **45**, 22–29. (doi:10.1016/j.jtherbio.2014.07.005)

3.	Schwarzkopf L, Alford RA. 1996 Desiccation and shelter-site use in a tropical amphibian: Comparing toads with physical models. *Funct Ecol* **10**, 193–200.

4.	Navas CA, Arauja C. 2000 The use of agar models to study amphibian thermal ecology. *J Herpetol* **34**, 330–334.

5.	Voyles J, Johnson LR, Rohr J, Kelly R, Barron C, Miller D, Minster J, Rosenblum EB. 2017 Diversity in growth patterns among strains of the lethal fungal pathogen *Batrachochytrium dendrobatidis* across extended thermal optima. *Oecologia* **184**, 363–373. (doi:10.1007/s00442-017-3866-8)

6.	Boyle D, Boyle D, Olsen V, Morgan J, Hyatt A. 2004 Rapid quantitative detection of chytridiomycosis (*Batrachochytrium dendrobatidis*) in amphibian samples using real-time Taqman PCR assay. *Dis Aquat Organ* **60**, 141–148. (doi:10.3354/dao060141)

7.	Hyatt A *et al.* 2007 Diagnostic assays and sampling protocols for the detection of *Batrachochytrium dendrobatidis*. *Dis Aquat Organ* **73**, 175–192. (doi:10.3354/dao073175)

8.	Allender MC, Bunick D, Mitchell MA. 2013 Development and validation of TaqMan quantitative PCR for detection of frog virus 3-like virus in eastern box turtles (*Terrapene carolina carolina).* *J Virol Methods* **188**, 121–125. (doi:10.1016/j.jviromet.2012.12.012)

9.	Leung WTM, Thomas-Walters L, Garner TWJ, Balloux F, Durrant C, Price SJ. 2017 A quantitative-PCR based method to estimate ranavirus viral load following normalisation by reference to an ultraconserved vertebrate target. *J Virol Methods* **249**, 147–155. (doi:10.1016/j.jviromet.2017.08.016)

10.	Picco AM, Brunner JL, Collins JP. 2007 Susceptibility of the endangered California Tiger Salamander*, Ambystoma californiense*, to ranavirus infection. *J Wildl Dis* **43**, 286–290. (doi:10.7589/0090-3558-43.2.286)

11.	Standish I, Leis E, Schmitz N, Credico J, Erickson S, Bailey J, Kerby J, Phillips K, Lewis T. 2018 Optimizing, validating, and field testing a multiplex qPCR for the detection of amphibian pathogens. *Dis Aquat Organ* **129**, 1–13. (doi:10.3354/dao03230)

12.	Longo A V., Rodriguez D, da Silva Leite D, Toledo LF, Mendoza Almeralla C, Burrowes PA, Zamudio KR. 2013 ITS1 Copy number varies among *Batrachochytrium dendrobatidis* strains: Implications for qPCR estimates of infection intensity from field-collected amphibian skin swabs. *PLoS One* **8**, e59499. (doi:10.1371/journal.pone.0059499)

13.	Hammond TT, Curtis MJ, Jacobs LE, Gaffney PM, Clancy MM, Swaisgood RR, Shier DM. 2021 Overwinter behavior, movement, and survival in a recently reintroduced, endangered amphibian, *Rana muscosa*. *J Nat Conserv* **64**. (doi:10.1016/j.jnc.2021.126086)

14.	Hammond TT, Backlin AR, Gallegos EA, Shier DM, Swaisgood RR, Fisher RN. 2025 Understanding and predicting infection dynamics for an endangered amphibian using long-term surveys of wild and translocated frogs. *Biol Conserv* **301**, 110834. (doi:10.1016/j.biocon.2024.110834)
